# A Nomogram Model Based on Noninvasive Bioindicators to Predict 3-Year Risk of Nonalcoholic Fatty Liver in Nonobese Mainland Chinese: A Prospective Cohort Study

**DOI:** 10.1155/2020/8852198

**Published:** 2020-11-02

**Authors:** Xintian Cai, Xiayire Aierken, Ayguzal Ahmat, Yuanyuan Cao, Qing Zhu, Ting Wu, Nanfang Li

**Affiliations:** Hypertension Center of People's Hospital of Xinjiang Uygur Autonomous Region, Xinjiang Hypertension Institute, National Health Committee Key Laboratory of Hypertension Clinical Research, Urumqi, China

## Abstract

The purpose of this study is to establish and validate an accurate and personalized nonalcoholic fatty liver disease (NAFLD) prediction model based on the nonobese population in China. This study is a secondary analysis of a prospective study. We included 6,155 nonobese adults without NAFLD at baseline, with a median follow-up of 2.3 years. Univariate and multivariate Cox regression analyses were used to determine independent predictors. The least absolute shrinkage and selection operator (LASSO) regression analysis was used to optimize the selection of variables. Based on the results of multivariate analysis, a prediction model was established. Harrell's consistency index (C-index) and area under the curve (AUC) were used to determine the discrimination of the proposed model. The goodness of fit of the calibration model was tested, and the clinical application value of the model was evaluated by decision curve analysis (DCA). The participants were randomly divided into a training cohort (*n* = 4,605) and a validation cohort (*n* = 1,550). Finally, seven of the variables (HDL-c, BMI, GGT, ALT, TB, DBIL, and TG) were included in the prediction model. In the training cohort, the C-index and AUC value of this prediction model were 0.832 (95% confidence interval (CI), 0.820-0.844) and 0.861 (95% CI, 0.849-0.873), respectively. In the validation cohort, the C-index and AUC values of this prediction model were 0.829 (95% CI, 0.806-0.852) and 0.859 (95% CI, 0.841-0.877), respectively. The calibration plots demonstrated good agreement between the estimated probability and the actual observation. DCA demonstrated a clinically effective predictive model. Our nomogram can be used as a simple, reasonable, economical, and widely used tool to predict the 3-year risk of NAFLD in nonobese populations in China, which is helpful for timely intervention and reducing the incidence of NAFLD.

## 1. Introduction

Nonalcoholic fatty liver disease (NAFLD) is a metabolic stress liver injury closely related to insulin resistance (IR) and genetic susceptibility [1]. NAFLD is the most common chronic liver disease in the world, and the prevalence of NAFLD in ordinary adults ranges from 6.3% to 45%. The prevalence of NAFLD in the Middle East and South America is the highest and the lowest in Africa [2]. The prevalence of NAFLD in most Asian countries, including China, is at the middle to the upper level (>25%) [3, 4]. In the past decade, the clinical burden of NAFLD is not limited to liver-related morbidity and mortality, and increasingly evidence shows that NAFLD is also closely related to the high incidence of metabolic syndrome (Mets), type 2 diabetes, arteriosclerotic cardiovascular disease, chronic kidney disease, and colorectal tumor [5–7]. With the prevalence of obesity and Mets, NAFLD has become the largest chronic liver disease in China [8]. Although NAFLD is more prevalent in obese people, nonobese NAFLD patients are not uncommon [9]. Epidemiological data show that 10%-30% of nonobese individuals have evidence of hepatic steatosis nonobese NAFLD [10, 11]. It is worth noting that nonobese NAFLD appears to be more common in Asians than in other populations [11]. In addition, nonobese NAFLD individuals may represent a subset of NAFLD in metabolically obese but normal-weight individuals, and their metabolic abnormalities are similar to those associated with obesity [12]. These patients showed high incidence rate of cardiovascular disease, diabetes, and all-cause mortality, which makes it an enormous health burden [13–15]. The causes are various and not completely understood, but NAFLD is reversible in the early stages. Despite the lack of pharmacological therapy, there are effective lifestyle interventions such as dietary changes, increased physical activity, and energy restriction [16]. These interventions are particularly effective in the early stages of the disease. Therefore, identifying high-risk nonobese patients and managing their metabolic status should be a key public health priority. The current challenge is how to identify these high-risk groups.

At present, there is no published NAFLD risk prediction model suitable for the nonobese Chinese population based on a prospective design. Liver biopsy is still the gold standard for diagnosis, but its disadvantages are its high cost, invasiveness, and many complications [17, 18]. In contrast, ultrasonography is a noninvasive method that has been widely used in the diagnosis of NAFLD [19]. However, in rural areas and remote areas, ultrasonography is inconvenient and too expensive for routine health examinations and screening in a large population. In view of these inherent limitations of imaging modalities and liver biopsy, in recent years, more and more attention has been paid to the possibility of evaluating NAFLD by using noninvasive clinical variables that can be measured in peripheral blood [2, 20]. Therefore, a few previous studies have focused on the establishment of NAFLD risk prediction model with noninvasive measures [20, 21]. The most commonly used variables in these models are biochemical indicators, including alanine transferase (ALT), total cholesterol (TC), and high-density lipoprotein cholesterol (HDL-c), but most predictive models include one or two biomarkers not included in a routine health examination, such as serum *α*2-macroglobulin, hyaluronic acid, and insulin levels [2]. Additionally, an ideal noninvasive test should be low cost, easy to obtain, simple and effective, and would make the detection and identification of NAFLD high-risk groups more intuitive. With such a test, it would be possible to carry out large-scale population screening and prevention programs in a large population. Therefore, in this study, we developed and validated an accurate personalized prediction model of NAFLD. The model takes the nonobese Chinese population as the research object, uses cost-effectiveness and easily accessible parameters to establish an accurate and individualized prediction model of NAFLD, to better assess the 3-year risk of NAFLD.

## 2. Materials and methods

### 2.1. Data Source

The data in the study comes from the Dryad digital repository website (http://www.datadryad.org), allowing users to download raw data for free. The data is anonymous. According to Dryad's terms of service, researchers can use these data for secondary analysis without infringing on the author's rights. In this study, the data came from the following sources: “Association of Low-Density Lipoprotein Cholesterol within the Normal Range and NAFLD in the Non-obese Chinese Population: A Cross-Sectional and Longitudinal Study.” Dataset website is 10.5061/dryad.1n6c4. We cited Dryad data package in the present study (Dryad data package: Dan-Qin Sun, Sheng-Jie Wu, Wen-Yue Liu, Li-Ren Wang, Yi-Ran Chen, Dong-Chu Zhang, Martin Braddock, Ke-Qing Shi, Dan Song, and Ming-Hua Zheng (2018) Data from “Association of Low-Density Lipoprotein Cholesterol within the Normal Range and NAFLD in the Non-obese Chinese Population: A Cross-Sectional and Longitudinal Study.” Dryad Digital Repository. doi:10.1136/bmjopen-2016-013781) [22].

### 2.2. Study Design and Population

It is worth noting that Dan-Qin Sun and his collaborators completed the entire study. To give readers a clear understanding of the design and implementation steps of the entire study, we briefly recapitulate this point. All participants participated in a health examination at the Wenzhou Medical Center of Wenzhou People's Hospital from January 2010 to December 2014. This prospective cohort study initially enrolled 33,153 subjects. Dan-Qin Sun and his collaborators developed the following exclusion criteria: (1) excessive alcohol consumption (male > 140 g/week, female > 70 g/week); (2) had a history of viral hepatitis, autoimmune hepatitis, or other known causes of chronic liver disease; (3) body mass index (BMI) ≥ 25 kg/m^2^; (4) low − density lipoprotein cholesterol (LDL − c) > 3.12 mmol/L; (5) were taking antidiabetic agents, lipid-lowering agents, or antihypertensive agents; and (6) loss of follow-up or lack of data. Since not all individuals met the criteria, in the end, a total of 16,173 nonobese individuals who initially did not have NAFLD were included and completed a 5-year follow-up examination. On the basis of the original exclusion criteria, this study formulated more stringent exclusion criteria. If any of the following conditions were met, participants were not included in this study: no specific follow-up time, no available gender information, no available weight and height measurements, no available blood pressure values, no available alkaline phosphatase (ALP), no *γ*-glutamyl transpeptidase (GGT), no ALT, no aspartate aminotransferase (AST), no total protein (TP), no albumin (ALB), no globulin (GLB), no total bilirubin (TB), no direct bilirubin (DBIL), no blood urea nitrogen (BUN), no creatinine (Cr), no uric acid (UA), no LDL-c, no fasting blood glucose (FPG), no HDL-c, no TC, and no triglycerides (TG). Finally, a total of 1688 baseline participants were obtained. Annual follow-up evaluations were conducted for all subjects during the observation period. The procedure for follow-up assessment was the same as at baseline. The secondary analysis was approved by the People's Hospital of Xinjiang Uygur Autonomous Region (Urumqi, China). Since the data used in this study comes from public databases and the patient information is anonymous, informed consent is not required. See the original report for details.

### 2.3. Variable Collection

Variables of each case from the raw data were extracted as follows: gender, age, BMI, GGT, ALT, AST, TP, ALB, GLB, TB, DBIL, BUN, Cr, EGFR, UA, LDL-c, FPG, HDL-c, TC, TG, fatty liver, and follow-up time. In short, medical history and health habit questionnaires were conducted by senior physicians. BMI (kg/m^2^) was used as an index of body fat, calculated by dividing body weight (kg) by height (m^2^). In a quiet environment, the participants sat and measured their blood pressure with an automatic sphygmomanometer. All laboratory indicators were measured by automatic analyzer (Abbott AxSYM) using standard methods.

### 2.4. Definitions of NAFLD

The ultrasound diagnostic standards for fatty liver were based on the standards proposed by the Chinese Liver Disease Association [22, 23]. NAFLD was defined as diffuse enhancement of liver near-field echo (stronger than the kidney and spleen area), far-field echo gradually weakening, and must be combined with one of the following conditions: (1) unclear intrahepatic lacunar structure, (2) mild to moderate hepatomegaly with blunt boundary, (3) unclear or incomplete right hepatic lobe and diaphragm capsule, and (4) decreased blood flow signal but normal blood flow distribution [22, 23]. The diagnosis of NAFLD is abdominal ultrasonography performed by trained technicians.

### 2.5. Statistical Analysis

Statistical analysis was performed using the R software version 3.6.1 (R Foundation for Statistical Computing, Vienna, Austria). When comparing the baseline characteristics of the development group and the validation group, the continuous variables of normal distribution were expressed as mean ± standard deviation. For those indicators with obvious skew distribution, their characteristics were described by median (1st quartile, 3rd quartile), and the categorical variables were expressed by frequency (proportion). The mean values of continuous variables were compared by independent group *t*-test of normal distribution data and Mann–Whitney test of nonnormal distribution data. Chi-squared test or Fisher's exact test was used to compare the categorical variables. The levels of ALT, GGT, TB, DBIL, and TG showed a positively skewed distribution. After logarithmic (Lg10) transformation, the data of ALT, GGT, TB, DBIL, and TG were all approximately normally distributed with geometric mean and corresponding 95% confidence interval (CI).

To improve the robustness and reliability of our conclusions, 6,155 NAFLD patients were randomly divided into a training cohort with 4,605 participants and a validation cohort with 1,550 participants at a ratio of 7.5 : 2.5 using R caret package, which met the theoretical ratio of 3 : 1.

The theory of nomogram was put forward by French engineer Philbert Maurice d'Ocagne in 1884 [24]. In the field of medicine, the advantage of nomogram is that it can personally predict a certain clinical outcome or the probability of a certain type of event, so it has great value in clinical practice [25]. To test and validate the prediction accuracy of the nomogram model, the training cohort and validation cohort were fully discriminated against and calibrated, respectively. The discrimination was evaluated by Harrell's consistency index (C-index). The index was similar to the area under the receiver operating characteristic (ROC) curve (AUC), and the absolute value is close to 1, indicating that the model has a strong prediction ability. Calibration refers to the consistency between the predicted risk and the actual risk, which was calculated by the Hosmer-Lemeshow test and visualized by calibration plot. A well-fitted model was not significant on the Hosmer-Lemeshow test, indicating that the model was not significantly different from the actual prediction. Decision curve analysis (DCA) was used to evaluate the clinical usefulness of nomograms. The net benefit was calculated by subtracting the proportion of patients with false positive results from the proportion of patients with real positive results and weighing the relative risk of intervention with the adverse effects of unnecessary intervention. 1,000 bootstrap resamples were applied to the C-index, AUC value, and calibration curve.

## 3. Results

### 3.1. Characteristics of Study Participants

According to the raw data provided by Dan-Qin Sun et al., a total of 16,173 nonobese patients without NAFLD were included in the study. After data processing, the patients with missing basic information and incomplete detection indicators were eliminated, and finally, 6,155 valid data were obtained. Eligible participants were randomly divided into training cohort (*n* = 4,605) and validation cohort (*n* = 1,550). In the training cohort, the average age was 45.74 years; 2,572 (55.85%) were male, and 19.93% of the participants (*n* = 918) were diagnosed with NAFLD at the end of follow-up. In the validation cohort, the average age was 45.74 years; 850 (54.84%) were male, and 19.61% of the participants (*n* = 304) were diagnosed with NAFLD at the end of follow-up. The average follow-up period of the training and validation datasets was 831 days and 825 days, respectively. There was no significant difference in baseline characteristics between training cohort and validation cohort (Table 1). The baseline characteristics of training cohort stratified according to incidence rate of NAFLD are shown in Table 2.

### 3.2. Feature Selection by LASSO Method

**Figure 1:**
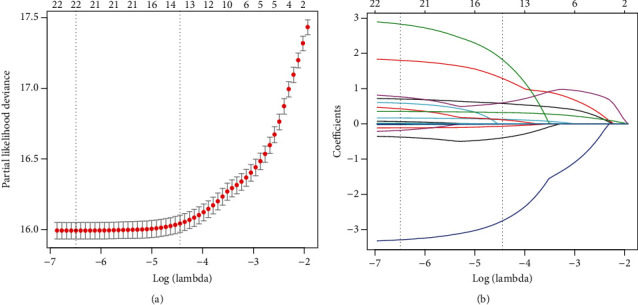
Clinical feature selection using the LASSO regression analysis with tenfold cross-validation. (a) Selection of optimal parameters (lambda) from the LASSO model using 10-fold cross-validation and minimum criteria. The partial likelihood deviance (binomial deviance) curve was plotted versus log (lambda). Dotted vertical lines were drawn at the optimal values using the minimum criteria and the 1 standard error of the minimum criteria (1-SE criteria). (b) LASSO coefficient profiles of the 22 features. A vertical line was drawn at the value selected using 10-fold cross-validation, where the best lambda resulted in 13 features with nonzero coefficients.

Based on the analysis of the results of the questionnaire survey, 22 variables were selected from demographic characteristic index, body mass index, and biochemical indicators to be included in the least absolute shrinkage and selection operator (LASSO) regression analysis (Figures 1(a) and 1(b)).  Figure 1(a) shows the LASSO model's 10-fold cross-validation error rate and the number of selected variables at grid values of *λ* (logarithmic scale). The most parsimonious and regularized model with a tuning *λ* (logarithmic scale) giving an error within 1 standard error of the minimum, included 13 variables (Lg (GGT), Lg (ALT), Lg (TB), Lg (DBIL), Lg (TG), Age, AST, BUN, UA, HDL-c, TC, BMI, and DBP).  Figure 1(b) shows the path of all candidate variable coefficients included in the model according to the level of logarithmic transformation *λ*.

### 3.3. Univariate and Multivariate Analysis in the Training Cohort

Univariate analyses showed that Age, ALP, AST, TP, ALB, BUN, Cr, UA, FPG, TC, HDL-c, LDL-c, BMI, SBP, DBP, Lg (GGT), Lg (ALT), Lg (TB), Lg (DBIL), and Lg (TG) were significantly different between NAFLD and non-NAFLD patients. Multivariate Cox regression analyses showed that Lg (GGT) (relative risk (HR) 1.96; 95% CI 1.51-2.53), FPG (HR 1.17; 95% CI 1.11-1.22), HDL-c (HR 0.72; 95% CI 0.55-0.92), LDL-c (HR 1.63; 95% CI 1.31-2.02), BMI (HR 1.43; 95% CI 1.37-1.49), Lg (ALT) (HR 5.23; 95% CI 3.52-7.78), Lg (TB) (HR 9.35; 95% CI 5.86-14.93), Lg (DBIL) (HR 0.06; 95% CI 0.05-0.08), and Lg (TG) (HR 2.62; 95% CI 1.74-3.93) were independent risk factors for NAFLD patients in the Chinese population (Table 3).

### 3.4. Predictive Model Construction

**Figure 2:**
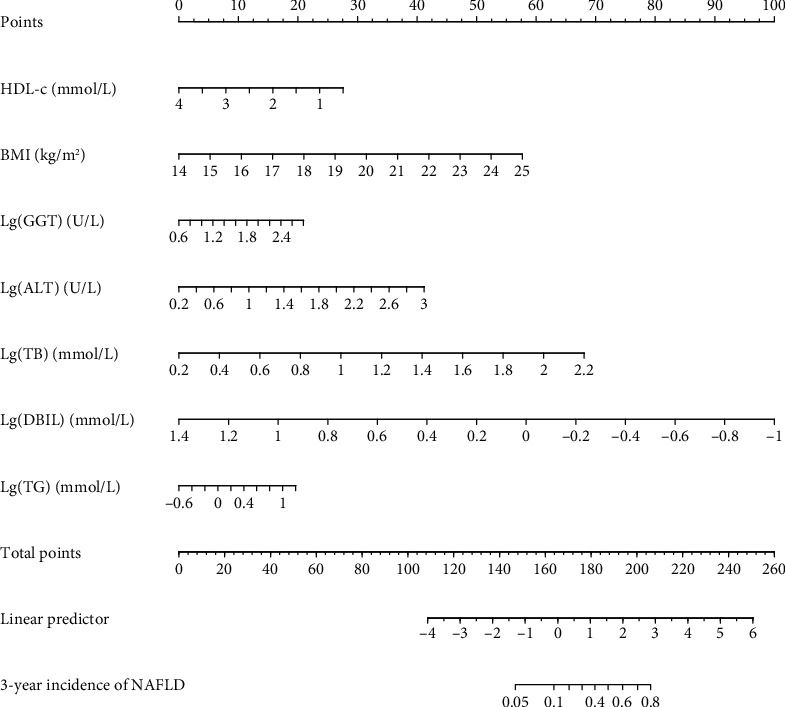
A nomogram for predicting the 3-year risk probability of NAFLD in the Chinese population. The nomogram is used by scoring on the scoring scale corresponding to each variable. ^∗^Instructions: to estimate an individual's 3-year risk of NAFLD, locate his/her value on each variable axis. Draw a vertical line from this value to the top point scale to determine how many points are assigned by that variable value. Then, the points of each variable value are added. Position the sum on the total point scale and project it vertically on the lower axis to obtain the personalized 3-year risk of NAFLD for the Chinese population.

Through the screening of LASSO regression and multivariate Cox regression analysis, the final prediction model established includes HDL-c, BMI, Lg (GGT), Lg (ALT), Lg (TB), Lg (DBIL), and Lg (TG) as predictors. The prediction model is presented in the form of a nomogram, which is used to quantitatively predict the 3-year risk probability of NAFLD in Chinese population (Figure 2). To estimate an individual's 3-year risk of NAFLD, his/her value is located on each variable axis. Draw a vertical line from the value to the top point scale to determine how many points the variable value specifies. Then, the points of each variable value are summed. The sum is located on the total point scale and projected vertically on the lower axis, thus obtaining the personalized 3-year risk of NAFLD.

### 3.5. Model Performance for Training and Validation Cohort

**Figure 3:**
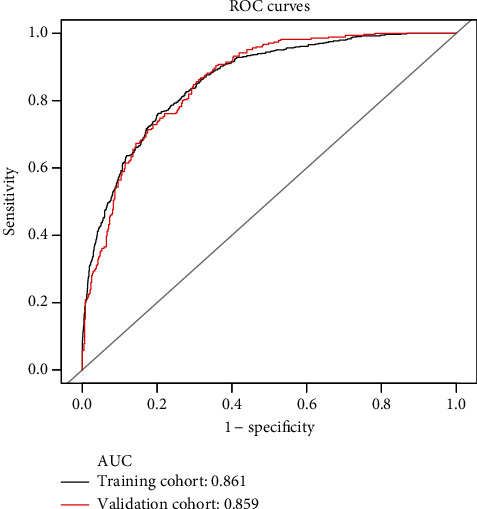
The ROC curves of the nomogram for 3-year NAFLD risk in the training and validation cohort. It shows that the AUC achieves 0.861 (95% CI, 0.849-0.873) and 0.859 (95% CI, 0.841-0.877) in the training cohort (black line) and the validation cohort (red line), respectively. ROC: receiver operating characteristics curves; AUC: area under curve; CI: confidence interval. ^∗^Using bootstrap resampling (times = 1000).

**Figure 4:**
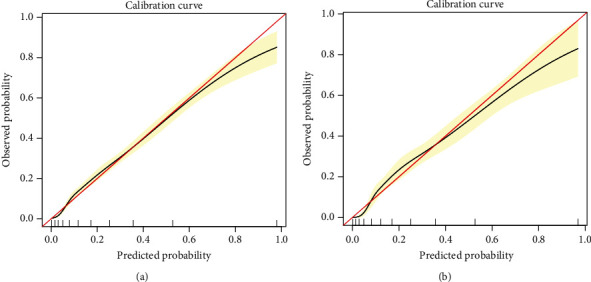
Calibration curves for the training and validation cohort models: (a) calibration curve of the model in the training cohort; (b) calibration curve of the model in the validation cohort. The red solid line represents a perfect prediction by an ideal model, and the solid black line shows the performance of the model. The yellow shadow represents the 95% confidence interval. ^∗^Using bootstrap resampling (times = 1000).

**Figure 5:**
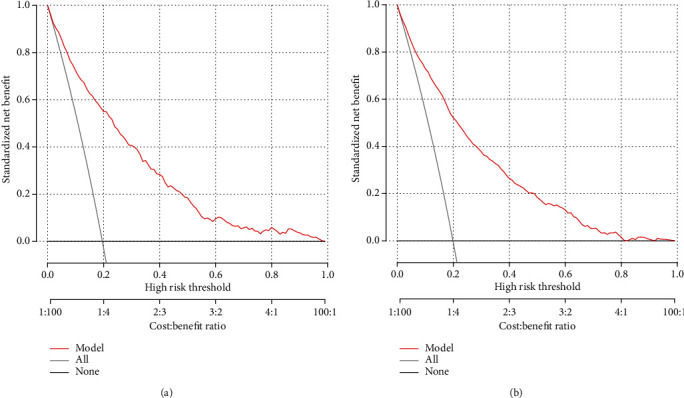
The DCA curve of the nomogram in the training and validation cohort: (a) the DCA of the nomogram for 3-year NAFLD risk in the training cohort; (b) the DCA of the nomogram for 3-year NAFLD risk in the validation cohort. The red solid line is the prediction model; the gray solid line is all NAFLD patients, and the solid line horizontal line is the non-NAFLD patients. The graph depicts the expected net benefit of each patient relative to nomogram in predicting NAFLD risk. With the extension of the model curve, the net benefit increases. ^∗^Using bootstrap resampling (times = 1000).

The C-index and AUC value were used to evaluate the discriminative ability of the prediction model. As a result, the model was validated internally by 1,000 bootstrap resamples. In the training cohort, the C-index and AUC value of this prediction model were 0.832 (95% CI, 0.820-0.844) and 0.861 (95% CI, 0.849-0.873), respectively (Figure 3). In the validation cohort, the C-index and AUC values of this prediction model were 0.829 (95% CI, 0.806-0.852) and 0.859 (95% CI, 0.841-0.877), respectively (Figure 3). This shows that the discriminative ability of this prediction model is quite good. Calibration curve and Hosmer-Lemeshow test were used to correct the prediction model. The calibration curve (Figure 4) shows a good agreement between the actual probability and the predicted probability. As shown in the Hosmer-Lemeshow test, the predicted and actual probabilities are highly consistent (training cohort, *P* = 0.845; validation cohort, *P* = 0.671). Next, we perform DCA on the nomogram in the training cohort and validation cohort, as shown in Figure 5. The DCA shows that the net benefit of the prediction model is significantly higher than that of the two extreme cases, whether in the training cohort or in the validation cohort. In general, the DCA shows that the nomogram is feasible and can make valuable and useful judgments.

## 4. Discussion

In recent years, NAFLD is not only common in developed countries but also in developing countries, so it is a global rather than regional public health problem [26]. NAFLD is a liver manifestation of Mets, and its potential cause seems to be hyperlipidemia. NAFLD can increase the risk of other liver diseases, including nonalcoholic steatohepatitis (NASH) cirrhosis and NASH hepatocellular carcinoma [27]. At present, numerous studies have shown that obesity is a well-known risk factor for NAFLD, while the relationship between nonobese population and NAFLD is often ignored [28, 29]. Some studies have shown that nonobese NAFLD is likely a different entity than obese NAFLD, with its unique genetic predisposition [30]. Moreover, nonobese NAFLD is more closely related to the components of metabolic syndrome [31]. A large number of studies have pointed out that primary prevention and timely intervention are the core of preventing or delaying the onset of NAFLD, whether obese or nonobese [32, 33]. Lifestyle changes in primary prevention, including eating habits and physical activity, are and should be the first treatment for people at high risk of NAFLD [27]. In general, any form of healthy diet (low fat or low carbohydrate or Mediterranean diet) should be encouraged, which will lead to reduced calories and be acceptable to patients [33]. For those who believe that calorie restriction is difficult, changing diet without necessarily reducing calorie intake may be a more viable option, although the benefit to liver health is not as significant as reducing calorie intake. Exercise produces significant but modest changes in liver fat (compared with reduced calorie intake) [34, 35]. However, considering the great cardiovascular benefits of exercise, the optimal placement for exercise may be used as an adjunct to dietary manipulation [36, 37]. Heavy drinking is closely related to the disease progression of NAFLD. For people at high risk of NAFLD, large amounts of alcohol should be avoided as much as possible (i.e., >4 drinks on a given day or >14 drinks per week for men and > 3 drinks on a given day or > 7 drinks per week for women) [38, 39]. Therefore, early detection of those at high risk of NAFLD is essential to reduce the incidence, which prompted us to conduct this study.

In this population-based cohort study, we developed a simple and quantifiable nomogram to predict the 3-year risk of NAFLD in Chinese nonobese population. To our knowledge, our study is the first to develop a nomogram for predicting the 3-year risk of NAFLD in nonobese populations in China. In our study, the raw data was randomly divided into a training cohort (*n* = 4,605) and a validation cohort (*n* = 1,550). Great degrees of discrimination and prediction ability were found both in the training cohort (AUC = 0.861) and the validation cohort (AUC = 0.859), which indicated that there was a relatively good predictive ability to distinguish individuals who are at risk to develop NAFLD from those who are not. The calibration curve shows that the constructed nomogram is accurate for predicting the risk of NAFLD. In addition, the decision curve analysis showed that nomogram could avoid liver ultrasound examination for individuals with low risk of NAFLD within 3 years, reducing the burden and cost.

Our prediction model includes HDL-c, BMI, Lg (GGT), Lg (ALT), Lg (TB), Lg (DBIL), and Lg (TG). These variables identified as risk factors for NAFLD were consistent with previous studies. In our prediction model, BMI is one of the main aspects of NAFLD risk factor scores. A large number of studies have shown that overweight or obesity is a well-known risk factor for NAFLD [29, 32, 40]. In obesity-induced metabolic disorders, lipid metabolism process changes, and fat organ dysfunction plays an important role in the occurrence of NAFLD [1, 29]. In this study, we found that even nonobese individuals (BMI < 25 kg/m^2^) had an increased risk of NAFLD with the increase of BMI.

According to previous studies, dyslipidemia is a well-known risk factor for NAFLD [41]. In particular, high TG levels and low HDL-c levels play an important role in the existence, development, and regression of NAFLD in nonobese individuals [41, 42]. In the nonobese population, the mechanism between high TG levels, low HDL-c levels, and NAFLD has not been fully explained, but IR is a potential mediated factor [10]. First of all, IR is closely related to NAFLD in nonobese population, and TG/HDL-c can be used as an independent predictor of IR [10]. At high TG levels, free fatty acids (FFAs) increase as lipolysis improves. Increased levels of FFAs can lead to deterioration of insulin sensitivity, and the induction of tissue oxidative stress can lead to tissue IR [43]. On the other hand, IR promotes the synthesis of triglycerides in the liver of NAFLD by inducing TG in adipose tissue and nascent lipolysis [44].

A large number of studies have shown that ALT and GGT are independent predictors of NAFLD [45, 46]. IR, mitochondrial dysfunction, increased production of proinflammatory cytokines, and oxidative stress lead to hepatocyte destruction/damage, which are considered the important pathophysiological mechanisms of NAFLD [47]. An elevated level of ALT, a glycogen enzyme synthesized in the liver, has been shown to be an indicator of impaired insulin signaling and develops hepatic IR [48]. On the other hand, serum GGT, a hepatobiliary enzyme synthesized in intrahepatic duct epithelial cells, closely related to hepatic steatosis and considered as a surrogate marker of NAFLD [49]. The underlying mechanisms of hepatic steatosis induced by elevated GGT have not been clearly defined. Ortega et al. proposed that the increase of liver fat deposition leads to hepatocyte injury and simulates the synthesis of GGT [50]. These increased levels of GGT enhance free radicals and mitochondrial damage, which can cause severe proinflammation and oxidative stress. As a surface enzyme, GGT can cleave extracellular glutathione (GSH), maintain the balance of GSH in *vivo*, and play a key role in alleviating the effects of oxidative stress. GGT is a main thiol antioxidant agent in mammalian cells. The increased of GGT level can induce GSH to hydrolyze to cysteinylglycine, then oxidized to produce reactive oxygen species, and induce mild hepatitis through hepatic steatosis. Recent studies have shown that GGT may be a reliable and simple marker of visceral and hepatic fat deposition and that hepatic fat denaturation can lead to hepatic IR, which can lead to metabolic abnormalities in the long term [47, 51].

A number of prospective cohort studies have shown that DBIL levels are significantly associated with a reduction in the risk of NAFLD, providing a protective biomarker for NAFLD [52]. More importantly, this association is independent of classic risk factors including liver enzymes, coronary heart disease, Mets, diabetes, and other classic metabolic risk factors [53]. However, the relationship between TB and indirect bilirubin and the risk of NAFLD was not significant. This may be partly due to DBIL, which is more soluble in serum and acts in an active form before indirect bilirubin [52, 53]. This conflicts with our research results. This contradiction may be caused by the following reasons: first, there are differences in the sources of participants among different studies. Secondly, there are also large differences in sample sizes between different studies. Finally, there are differences in the methods of TB testing between different studies. The biological mechanism of negative correlation between DBIL and NAFLD risk has not been fully elucidated [54]. There is increasing evidence that oxidative stress is considered to be an inducement from benign steatosis to more advanced forms of NAFLD, and the reactive oxygen species produced by the oxidation of fatty acids are also considered a permanent factor in NAFLD liver damage [49]. It has been reported that bilirubin, the final product of haem catabolism, has been found to have potential antioxidant and cytoprotective effects in vitro and in *vivo*, which can antagonize oxidative stress [53]. In addition, another possible mechanism for linking bilirubin and NAFLD risk reduction is proposed by inhibiting IR. IR has been proven to be a recognized risk factor of NAFLD and a new biomarker of liver damage in NAFLD patients [55]. In fact, IR is also considered triggering the pathogenesis of NAFLD and oxidative stress are interdependent. Interestingly, recent evidence shows that elevated bilirubin has a protective effect on IR and significantly improves insulin sensitivity by upregulating adiponectin production and peroxisome proliferator-activated receptor levels [56]. In summary, these findings provide evidence that elevated bilirubin may contribute to the prevention of NAFLD by inhibiting IR and altering glucose metabolism. Finally, there is increasing evidence that bilirubin can reduce the risk of NAFLD by inhibiting inflammatory environment or complement activation and lipid accumulation, which has been frequently demonstrated to play an important role in the pathogenesis of NAFLD [57, 58]. It is reported that the anti-inflammatory effect of bilirubin plays an important role in reducing the production of proinflammatory cytokines (such as interleukin-6 and interleukin-1), which are involved in hepatic steatosis [59].

However, the current research has several potential limitations. First of all, the nomogram is based on a 3-year prospective study conducted in China. There are regional differences in the prevalence of NAFLD. Therefore, whether this nomogram model is applicable to other regions or countries needs further multicenter validation. Secondly, there may be differences in the normal value of clinical indicators and lifestyle among different ethnic groups, and the current study only includes the Han population in China. The model proposed here may not be applicable to the other ethnic groups or general population. Thirdly, this study lacks data on lifestyle, dietary factors, and physical activity indicators, so the prediction ability of the model is limited. Fourth, the study excluded individuals with incomplete data in the selection of participants, which may lead to selection bias. Fifthly, insulin content and IR cannot be detected in this study. IR may be closely related to NAFLD in nonobese individuals. Finally, the diagnosis of NAFLD is based on ultrasonography. In large-scale epidemiology and clinical practice, B-type ultrasonography is considered a widely accepted and cost-effective tool for screening NAFLD, which has reasonable accuracy and sensitivity for the detection of fatty liver. However, the use of ultrasound in the diagnosis of NAFLD will inevitably lead to the possibility of false negative and positive diagnosis, and the fact that the technique is highly operator-dependent. Although it was not reasonable to obtain a liver biopsy in all individuals, coupling ultrasonography with other parameters, such as homeostatic model assessment for insulin resistance, could enhance the strength of NAFLD diagnosis. Unfortunately, also these parameters were not available. Consequently, this study is able to predict the development of ultrasound-based NAFLD, rather than NAFLD properly.

## 5. Conclusion

In summary, we have established a nomogram based on seven risk factors including HDL-c, BMI, Lg (GGT), Lg (ALT), Lg (TB), Lg (DBIL), and Lg (TG). The nomogram developed in this study has been validated internally and can be used as a simple, reasonable, economical, and widely used tool to predict the 3-year NAFLD risk of nonobese population in China. The tool has the potential to be a cost-effective and noninvasive method to help clinicians identify high-risk groups and perform regular ultrasound examinations, take necessary measures for lifestyle monitoring, and medical interventions at an earlier stage, especially in primary health care centers. However, before the model can be widely used, it needs to be externally validated and modified for other populations.

## Figures and Tables

**Table 1:** Baseline clinical and laboratory characteristics of study population by training and validation cohort.

Characteristic	Training cohort	Validation cohort	*P* value
No. of participants	4,605	1,550	
Age (years)	45.74 ± 15.72	45.74 ± 15.57	0.992
ALP (U/L)	73.32 ± 25.20	72.30 ± 21.35	0.153
GGT (U/L)	22.00 (16.00-31.00)	22.00 (17.00-32.00)	0.287
ALT (U/L)	16.00 (12.00-22.00)	17.00 (12.00-23.00)	0.608
AST (U/L)	23.23 ± 9.76	23.68 ± 10.78	0.123
TP (U/L)	73.76 ± 4.40	73.76 ± 4.44	0.989
ALB (U/L)	44.39 ± 2.77	44.37 ± 2.63	0.723
GLB (U/L)	29.37 ± 4.18	29.40 ± 4.03	0.804
TB (mmol/L)	11.30 (8.90-14.50)	11.20 (8.90-14.30)	0.348
DBIL (mmol/L)	2.00 (1.50-2.80)	2.00 (1.40-2.70)	0.614
BUN (mmol/L)	4.72 ± 1.50	4.74 ± 1.64	0.534
Cr (*μ*mol/L)	84.57 ± 27.14	85.46 ± 35.15	0.304
UA (*μ*mol/L)	289.43 ± 88.62	292.53 ± 89.38	0.235
FPG (mmol/L)	5.31 ± 0.86	5.33 ± 1.03	0.45
TC (mmol/L)	4.63 ± 0.74	4.61 ± 0.78	0.231
TG (mmol/L)	1.14 (0.86-1.59)	1.14 (0.85-1.58)	0.793
HDL-c (mmol/L)	1.46 ± 0.36	1.45 ± 0.37	0.594
LDL-c (mmol/L)	2.29 ± 0.47	2.27 ± 0.49	0.249
BMI (kg/m^2^)	21.72 ± 2.02	21.68 ± 2.06	0.449
SBP (mmHg)	124.61 ± 17.79	124.90 ± 18.22	0.574
DBP (mmHg)	74.55 ± 10.49	74.35 ± 10.51	0.515
Gender (*n*, %)			0.487
Female	2033 (44.15%)	700 (45.16%)	
Male	2572 (55.85%)	850 (54.84%)	
Follow-up (days)	831.18 ± 376.22	825.26 ± 386.61	0.594
Incident NAFLD (*n*, %)			0.783
No	3687 (80.07%)	1246 (80.39%)	
Yes	918 (19.93%)	304 (19.61%)	

Data are *n* (%), mean ± SD, or median (interquartile range). ALP: alkaline phosphatase; GGT: *γ*-glutamyl transpeptidase; ALT: alanine aminotransferase; AST: aspartate aminotransferase; TP: total protein; ALB: albumin; GLB: globulin; TB: total bilirubin; DBIL: direct bilirubin; BUN: blood urea nitrogen; Cr: creatinine; UA: uric acid; FPG: fasting plasma glucose; TC: total cholesterol; TG: triglyceride; HDL-c: high-density lipoprotein cholesterol; LDL-c: low-density lipoprotein cholesterol; BMI: body mass index; SBP: systolic blood pressure; DBP: diastolic blood pressure; NAFLD: nonalcoholic fatty liver disease.

**Table 2:** Baseline characteristics of NAFLD and non-NAFLD patients in the training cohort.

Characteristics	Non-NAFLD	NAFLD	*P* value
No. of participants	3,687	918	
Age (years)	45.32 ± 15.71	47.42 ± 15.65	<0.001
ALP (U/L)	72.25 ± 25.78	77.62 ± 22.23	<0.001
Lg (GGT) (U/L)	1.35 ± 0.22	1.53 ± 0.27	<0.001
Lg (ALT) (U/L)	1.20 ± 0.20	1.35 ± 0.21	<0.001
AST (U/L)	22.78 ± 10.03	25.04 ± 8.34	<0.001
TP (U/L)	73.70 ± 4.33	73.99 ± 4.66	0.075
ALB (U/L)	44.35 ± 2.76	44.58 ± 2.79	0.024
GLB (U/L)	29.35 ± 4.07	29.41 ± 4.58	0.704
Lg (TB) (mmol/L)	1.05 ± 0.17	1.06 ± 0.16	0.142
Lg (DBIL) (mmol/L)	0.31 ± 0.23	0.26 ± 0.23	<0.001
BUN (mmol/L)	4.71 ± 1.54	4.75 ± 1.34	0.502
Cr (*μ*mol/L)	83.65 ± 28.21	88.27 ± 21.98	<0.001
UA (*μ*mol/L)	280.78 ± 87.46	324.19 ± 84.71	<0.001
FPG (mmol/L)	5.23 ± 0.78	5.60 ± 1.10	<0.001
TC (mmol/L)	4.60 ± 0.73	4.77 ± 0.75	<0.001
Lg (TG) (mmol/L)	0.24 ± 0.19	0.22 ± 0.22	<0.001
HDL-c (mmol/L)	1.50 ± 0.36	1.29 ± 0.31	<0.001
LDL-c (mmol/L)	2.26 ± 0.47	2.39 ± 0.48	<0.001
BMI (kg/m2)	21.36 ± 2.00	23.17 ± 1.33	<0.001
SBP (mmHg)	123.17 ± 17.77	130.39 ± 16.68	<0.001
DBP (mmHg)	73.47 ± 10.34	78.89 ± 9.94	<0.001
Follow-up (days)	868.02 ± 372.69	683.21 ± 353.47	<0.001
Gender (*n*, %)			0.227
Female	1644 (44.59%)	389 (42.37%)	
Male	2043 (55.41%)	529 (57.63%)	

Data are *n* (%) or mean ± SD. NAFLD: nonalcoholic fatty liver disease; ALP: alkaline phosphatase; GGT: *γ*-glutamyl transpeptidase; ALT: alanine aminotransferase; AST: aspartate aminotransferase; TP: total protein; ALB: albumin; GLB: globulin; TB: total bilirubin; DBIL: direct bilirubin; BUN: blood urea nitrogen; Cr: creatinine; UA: uric acid; FPG: fasting plasma glucose; TC: total cholesterol; TG: triglyceride; HDL-c: high-density lipoprotein cholesterol; LDL-c: low-density lipoprotein cholesterol; BMI: body mass index; SBP: systolic blood pressure; DBP: diastolic blood pressure.

**Table 3:** Results of univariate and multivariate analysis for NAFLD risk prediction.

Variables	Univariate analysis		Multivariate analysis	
	HR (95% CI)	*P* value	HR (95% CI)	*P* value
Gender				
Female	Reference			
Male	1.04 (0.92, 1.16)	NS		
Age	1.00 (1.00, 1.01)	0.012	0.99 (0.98, 1.01)	NS
ALP	1.01 (1.01, 1.01)	<0.001	1.00 (1.00, 1.00)	NS
AST	1.01 (1.00, 1.01)	<0.001	0.99 (0.97, 1.02)	NS
TP	1.02 (1.01, 1.03)	0.001	1.01 (0.99, 1.02)	NS
ALB	1.04 (1.02, 1.06)	<0.001	0.99 (0.97, 1.02)	NS
GLB	1.01 (0.99, 1.02)	NS		
BUN	0.89 (0.85, 0.93)	<0.001	0.90 (0.78, 1.02)	NS
Cr	1.00 (1.00, 1.00)	0.001	1.00 (1.00, 1.00)	NS
UA	1.00 (1.00, 1.00)	<0.001	1.00 (1.00, 1.00)	NS
FPG	1.23 (1.18, 1.28)	<0.001	1.17 (1.11, 1.22)	<0.001
TC	1.29 (1.20, 1.39)	<0.001	0.80 (0.57, 1.03)	NS
HDL-c	0.29 (0.25, 0.35)	<0.001	0.72 (0.55, 0.92)	0.010
LDL-c	1.69 (1.49, 1.91)	<0.001	1.63 (1.31, 2.02)	<0.001
BMI	1.64 (1.58, 1.71)	<0.001	1.43 (1.37, 1.49)	<0.001
SBP	1.01 (1.01, 1.01)	<0.001	0.99 (0.99, 1.00)	NS
DBP	1.03 (1.03, 1.04)	<0.001	1.00 (0.99, 1.01)	NS
Lg (GGT)	6.21 (5.25, 7.34)	<0.001	1.96 (1.51, 2.53)	<0.001
Lg (ALT)	7.82 (6.45, 9.47)	<0.001	5.23 (3.52, 7.78)	<0.001
Lg (TB)	0.60 (0.43, 0.85)	0.004	9.35 (5.86, 14.93)	<0.001
Lg (DBIL)	0.10 (0.08, 0.12)	<0.001	0.06 (0.05, 0.08)	<0.001
Lg (TG)	18.26 (14.65, 22.75)	<0.001	2.62 (1.74, 3.93)	<0.001

NAFLD: nonalcoholic fatty liver disease; ALP: alkaline phosphatase; GGT: *γ*-glutamyl transpeptidase; ALT: alanine aminotransferase; AST: aspartate aminotransferase; TP: total protein; ALB: albumin; GLB: globulin; TB: total bilirubin; DBIL: direct bilirubin; BUN: blood urea nitrogen; Cr: creatinine; UA: uric acid; FPG: fasting plasma glucose; TC: total cholesterol; TG: triglyceride; HDL-c: high-density lipoprotein cholesterol; LDL-c: low-density lipoprotein cholesterol; BMI: body mass index; SBP: systolic blood pressure; DBP: diastolic blood pressure; HR: hazard ratio; CI: confidence interval; NS: no significance.

## Data Availability

All datasets generated and/or analyzed during the present study are included in this published article and available in Dryad Digital Repository (http://www.datadryad.org/).
